# Everybody's Page

**Published:** 1888-04-14

**Authors:** 


### Everybody's Page.

Some deaf persons are acutely affected by vibratory impulses conveyed through the tissues of the body to the auditory nerve. Thus Kitto, the famous deaf mute, found that every movement or concussion in a wooden-floored, uncarpeted room caused him a sensation amounting to torture.

A most essential thing for the avoidance of deafness is to run no risk of catching cold in the head—perhaps its most common source. The ear is much more likely to be affected by a succession of slight attacks of cold than by a single severe one.

Milk is one of the best feeding and breeding grounds of those minute living organisms which poison the system ; and a quantity of impure water, which by itself might not amount to a poisonous dose, becomes very dangerous after it has been mixed with milk and allowed to remain for a time. It is a safe rule to boil all milk before using it.

It is rather a humiliating thing to be informed that the healthiest spots in her Majesty's dominions are the prisons, so that, so far as length of days goes, the best thing a decent citizen could do to secure freedom from the plagues and the "aches and thousand natural shocks that flesh is heir to," would be so to behave as to be taken under special State protection.

Man differs from all the other mammalia in the arrangement of his teeth. In him they are all of one uniform length, being all, generally speaking, on the same level at the crowns. Again, no interspaces exist among them, every tooth being close to the one next it. In the lower animals, on the other hand, empty spaces exist at certain parts along the jaw, the series of teeth being interrupted by gaps and vacancies, generally occurring near the eye-teeth. And the teeth themselves are not of uniform length, the eye-teeth in particular being almost always much longer than their neighbours, and overtopping them considerably.

The purest water, and that best adapted for general use in cleansing the skin, is undoubtedly distilled water, but at present it cannot be obtained at a reasonable price. Now, how ever, by the aid of a very simple but ingenious device, every householder possessing a boiler in his kitchen-range may obtain it for himself. A waste-pipe, commonly known among builders as a "blow-pipe," is attached to all kitchen boilers, and is carried into the chimney, where it serves as an outlet for the steam which is constantly being generated, and would otherwise burst the boiler. The steam, however, which is thus being wasted needs only to be captured, condensed, and retained in a suitable vessel, to provide us at once with an article which will not only effect a great saving in soap, but will render the cleansing of the skin a positive luxury.

People do not generally understand that the skin shares in the function of respiration, and that if this is interfered with death is apt to ensue, though this is due not so much to asphyxia as to some form of blood poisoning caused by the constituents of the perspiration being re-absorbed into the blood. A striking illustration of the necessity of leaving the skin open to some extent to the action of air is afforded by the fact that a child who was coated with gum and then covered with gold-leaf, to represent a cherub at the coronation of Pope Leo X., died a few hours after the operation. A similar fate nearly befell Gustave Doré in the days of his youth, when a passing caprice made him go to a fancy ball as "Un Monsieur Doré"; but as in his case the gilding was only partial, he survived, though he did not escape a sharp illness.

One may sometimes discover that a diseased or badly-stopped tooth, which is not in itself painful, is the cause of neuralgia in the external auditory canal.

Any occupation which necessitates severe and continued physical strain—as that of smiths, hammer-men, railway workers—is apt to bring on heart disease.

One dose of tea is quite sufficient in the twenty-four hours ; and over-indulgence in tea is the cause of innumerable headaches and so-called nervous diseases.

The use of soap in shaving is not, as is popularly supposed, to soften the hair, but, on the contrary, to render them hard, dry, stiff, and brittle. This is the condition in which they yield best to the razor.

In the Peabody model lodging-houses, where overcrowding is not permitted, the death-rate has been only 16·7 per thousand during the last sixteen years, while that of the crowded neighbouring district is from 30 to 40 per thousand.

The composition of milk varies slightly, according to the breed of the cows that yield it, the Alderneys giving more fat and the longhorns more caseine. As a rule it is the quantity, not the quality, of the milk that varies in different animals.

What is honey made from? The bees make it from the flowers, but man has much improved on this simple process. This is his method : He first makes a syrup from starch and oil of vitrol, and then adds a little genuine honey to give the compound the true flavour. After this the mixture is packed in neat little jars, furnished with a bright label, decorated perhaps with a picture of a bee-hive and its inhabitants, and sent into the market as honey.

Sharpness of vision differs considerably in different persons, and may be greatly improved by habits of attention ; but that it has not undergone material change for at least four thousand years is sufficiently attested by the fact that among the Arabs, whose pastoral habits, combined with the dryness and clearness of the atmosphere of their vast plains, led them to study the heavens at night, the small star Alcor, which is situated in the tail of the Great Bear, was regarded as a test of good vision. They named it Saidak, or the "Tester." It is still just within the limits of normal vision, and cannot be seen by the near-sighted.

What is a doctor's horse? This is not a conundrum, nor is it a question to be answered by the light of natural science, after this fashion—a doctor's horse is a mammalian quadruped, genus *equus*, and so forth. From the point of view of taxation, these things are of no account. The point is this—whether is a doctor's horse a trade or pleasure horse, an animal which should or should not be taxed. Is it to be classed with the squire's hunter or with the sturdy cob that draws the butcher's cart? If it is decided that dignity be sacrificed and the steed be looked on as part of a business establishment, he may go free ; but if its owner resents this classification, he must pay for his pride. The last suggestion made on the subject is that the doctor should be looked upon as a one-horse man, and if he keeps more he will be expected to submit to Mr. Goschen's tax. Probably most members of the profession will prefer to sacrifice pride to economy, and say the horse is kept for business purposes. But let us say a word of warning to the Frau Doctorinn. A trade horse must be kept strictly for trade, and if after the daily rounds are over she borrows the carriage to pay a few calls, she may chance thereby to bring the vindictive tax-gatherer, till then defrauded of his prey, down on her husband's head.

